# Recurrent Monosomies Confirmed by Interphase FISH in Three Chronic Myeloid Leukemia Cases

**DOI:** 10.4274/tjh.2014.0374

**Published:** 2015-02-15

**Authors:** Yelda Tarkan Argüden, Dilhan Kuru, Ayşe Çırakoğlu, Şükriye Yılmaz, Şeniz Öngören Aydın, Cem Muhlis Ar, Ayhan Deviren, Teoman Soysal, Seniha Hacıhanefioğlu

**Affiliations:** 1 İstanbul University Cerrahpaşa Faculty of Medicine, Department of Medical Biology, İstanbul, Turkey; 2 İstanbul University Cerrahpaşa Faculty of Medicine, Department of Internal Medicine, Division of Hematology, İstanbul, Turkey

**Keywords:** cytogenetics, Marrow, Neoplasia, Chronic myeloid leukemia, Monosomy

## TO THE EDITOR

Although chronic myeloid leukemia (CML) is characterized by the Philadelphia (Ph) chromosome, which is the result of t(9;22) (q34;q11) or its variants, 10%-20% of cases have additional cytogenetic abnormalities. The most common additional abnormalities are loss of the Y chromosome, +8, +Ph, and i(17q). Since these additional chromosome abnormalities are signs of disease progression, it is important to perform cytogenetic analyses periodically in patients with CML [1].

We have published our results on clonal chromosome abnormalities other than the Ph chromosome in Ph+ and Ph- cells of CML patients who were followed in our center a few years ago [2,3]. Monosomies were the most frequently observed chromosome abnormalities in these reports. In some cases, there were recurrent monosomies in more than one sample. To evaluate the significance of these recurrent monosomies, we performed fluorescence in situ hybridization (FISH) analysis for 3 of the previously reported patients with recurrent monosomies. Informed consent was obtained before the study. For FISH testing, chromosomes 8, 10, 17, and 20 were selected, since they had been the most common monosomies in our earlier publications.

All 3 patients in this study were under imatinib therapy except for patient 2, who was receiving interferon at the time of the first sampling. 

Conventional cytogenetic techniques were performed to examine the marrow samples. GTL-banded metaphases were examined according to International System for Human Cytogenetic Nomenclature guidelines [4]. Twenty metaphases were studied whenever possible. Cytocell Aquarius alpha satellite probes were used according to the manufacturer’s instructions for FISH. Two hundred interphase cells were counted by 2 different researchers. Normal karyotyped blood or marrow samples were used as control cases. Results of the cytogenetic and FISH analyses are given in Table 1.

In the first 3 samples of patient 1, monosomy of chromosomes 8, 10, and 20 was confirmed by FISH. In the fourth sample of this patient, we performed FISH for chromosomes 10 and 20 despite their absence in the karyotype to see whether there was a hidden monosomy that we could not show by karyotyping. FISH indeed revealed a hidden monosomy for chromosome 20, but not for chromosome 10. In the first 2 samples of patient 2, monosomy of chromosome 17 was observed in cytogenetic analysis and confirmed by FISH. However, in the third sample, -17 could be shown neither in the karyotype nor by FISH. Notably, percentages of -17 obtained by cytogenetics and FISH were compatible in each of the samples. In the first sample, -17 was detected cytogenetically in 3 out of 12 cells (25%), and it was found in 46% of the interphases by FISH. In the second sample, -17 was found in 12% and 31% of the cells in cytogenetic and FISH analysis, respectively. In the third, it was absent in cytogenetic preparations as well as in FISH study. -17 may be an important candidate marker to be followed by FISH in the course of CML since it is one of the additional minor-route chromosome abnormalities in the clonal evolution of Ph+ CML [1]. Furthermore, it leads to the loss of the p53 gene localized on 17p, which is known to be involved in CML progression [1]. In patient 3, -8 was confirmed by FISH, but -20 could not be demonstrated by FISH despite its presence in the karyotype.

FISH results were in line with those of cytogenetics in some samples while not in others. This, once again, highlights the importance of concurrent use of different techniques (i.e. FISH and conventional cytogenetics) in cancer samples to increase the detection capability and improve the reliability of the results. Studies with larger numbers of patient samples and longer follow-up are required to establish the impact of certain monosomies on the disease course.

## Figures and Tables

**Table 1:**
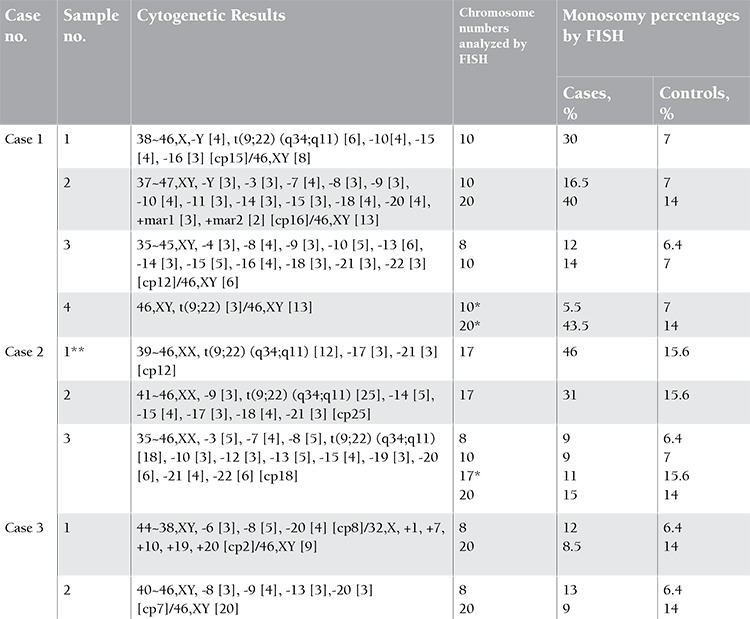
Cytogenetic and fluorescence in situ hybridization results.
